# Another Proposed Anæsthetic

**Published:** 1858-10

**Authors:** 


*Another proposed Anæsthetic.*—M. Herpin, of Metz, in a note addressed to the French Academy of Sciences, has suggested the employment of carbonic acid gas as an anæsthetic. He proposes to mix it with atmospheric air, and urges caution in its use, lest it should come in contact with the eyes and the nostrils. He also suggests the previous administration of chloroform, leaving the carbonic acid to complete the anæsthesia, having previously mixed it with eight or nine times its volume of atmospheric air. He thinks that by this means the danger of the exclusive use of chloroform will be avoided, since the mixture can be graduated at pleasure, and thus the exact amount of anæsthesia desirable in each case obtained. He considers it especially advantageous in indefinitely prolonging the duration of the anæsthesia.

We had rather let carbonic acid alone at present. Chloroform is quite dangerous enough for us, and the trouble about it is that the duration of its anæsthesia is rather too indefinite. Carbonic acid must be proved to be safer than chloroform before it can be used, and it must be admitted that it has no little prejudice against it to overcome. There is no doubt of its anæsthetic powers, but there is of its safety.

